# Enhancing Oral 5-ASA Effectiveness in Mild-to-Moderate Ulcerative Colitis through an *H. erinaceus*-Based Nutraceutical Add-on Multi-Compound: The “HERICIUM-UC” Two-Arm Multicentre Retrospective Study

**DOI:** 10.3390/pharmaceutics16091133

**Published:** 2024-08-28

**Authors:** Antonio Tursi, Alessandro D’Avino, Giovanni Brandimarte, Giammarco Mocci, Raffaele Pellegrino, Edoardo Vincenzo Savarino, Antonietta Gerarda Gravina, the HERICIUM-UC Study Group

**Affiliations:** 1 Territorial Gastroenterology Service, Barletta-Andria-Trani Local Health Agency, Via Fornaci, 76123 Andria, Italy; 2 Department of Medical and Surgical Sciences, Catholic University of Rome, Largo A. Gemelli, 00168 Roma, Italy; 3 Department of Internal Medicine, IRCCS Istituto Dermopatico dell’Immacolata, 00167 Roma, Italy; 4 Cristo Re Hospital, Via delle Calasanziane, 00167 Roma, Italy; 5 SC Gastroenterologia, ARNAS Brotzu, Piazzale A. Ricchi, 09047 Cagliari, Italy; 6 Hepatogastroenterology Division, Department of Precision Medicine, University of Campania Luigi Vanvitelli, Via L. de Crecchio, 80138 Napoli, Italy; 7 Gastroenterology Unit, Department of Surgery, Oncology and Gastroenterology, University of Padova, Via VIII Febbraio, 35121 Padova, Italy

**Keywords:** ulcerative colitis, nutraceuticals, *Hericium erinaceus*, 5-ASA, prebiotics, probiotics, berberine, quercetin, niacin, biotin

## Abstract

Mild-to-moderate ulcerative colitis (UC) management is centred on 5-aminosalicylic acid (5-ASA) derivatives. Whether supplementing 5-ASA with nutraceuticals can provide real advantages in UC-relevant outcomes is unclear. This retrospective multicentre study compared clinical remission, response rates, and faecal calprotectin levels in a two-arm design, including patients treated with 5-ASA alone and those with additional *H. erinaceus*-based multi-compound supplementation. In the 5-ASA alone group, clinical response rates were 41% at three months (T_1_) and 60.2% at six months (T_2_), while corresponding clinical remission rates were 16.9% and 36.1%. In the nutraceutical supplementation group, clinical response rates were 49.6% (T_1_) and 70.4% (T_2_), with clinical remission rates of 30.4% (T_1_) and 50.9% (T_2_). No significant differences in clinical response rates between the groups at T_1_ (*p* = 0.231) and T_2_ (*p* = 0.143) emerged. Clinical remission rates differed significantly at both time points (*p* = 0.029 and *p* = 0.042, respectively). Faecal calprotectin levels decreased significantly in both groups during the retrospective follow-up (*p* < 0.05), and this was more pronounced in nutraceutical supplementation patients at both T_1_ (*p* = 0.005) and T_2_ (*p* = 0.01). No adverse events were reported. This multi-component nutraceutical supplementation offers real-world potential in controlling disease activity in patients with mild-to-moderate UC.

## 1. Introduction

Ulcerative colitis (UC) is a relapsing–remitting inflammatory condition characterised by a chronic inflammatory process affecting the colorectal region, classified within the group of inflammatory bowel diseases [1].

The current medical management of UC follows a step-up approach that adjusts the intervention’s intensity to the severity of the disease. It involves conventional therapeutic measures, primarily consisting of 5-aminosalicylic acid (5-ASA) derivatives, topical and systemic steroids, and immunosuppressants such as azathioprine [2,3]. Additionally, advanced therapies are reserved for cases refractory to conventional treatment, steroid dependence, or significant disease severity, predominantly comprising biologics and small molecules [2].

Currently, the available evidence does not support a precise, systematic use and positioning of nutraceutical supplementation, especially pre- and probiotics, in the guidelines-driven therapeutic sequencing of UC. Recent and robust meta-analyses suggest uncertainty regarding their use in the induction and maintenance phases of remission [4,5]. In addition, it is not entirely clear whether such supplementation, when combined with 5-ASA therapy, can provide both temporal advantages (such as the speed of achieving the desired outcome) and quantitative benefits (such as the extent of clinical improvement) in UC management [5].

On the other hand, the therapeutic benchmark for UC is progressively rising, with consensus emphasising the need to aim higher, transitioning from achieving complete clinical, biochemical, endoscopic, and histological remission (i.e., deep remission) to a quality of life that entirely overlaps with that of the healthy general population (i.e., true “disease clearance”) [6]. The search for therapeutic agents, especially nutraceuticals, which can enhance the therapeutic efficacy of conventional or advanced treatments alongside an optimal safety profile, given their borderline status as functional foods [7], is desirable for achieving more favourable treatment outcomes.

Among the promising natural substances for UC is *Hericium erinaceus* (*H. erinaceus*), a fungus of Chinese origin. Pre-clinical studies have demonstrated its gastroprotective effects in repairing gastric mucosal damage, its antimicrobial properties against *Helicobacter pylori*, its anti-neoplastic effects against gastric and colorectal tumours (primarily through its components, erinacines), and its potential in inflammatory bowel diseases, including modulation of the intestinal microbiota acting as a pro- and prebiotic [8]. Similar beneficial pre-clinical results for UC have been observed using other natural substances, such as quercetin [9] and berberine [10]. Nonetheless, regular vitamin intake is crucial for UC, as indicated by the more pronounced biotin (i.e., vitamin B_8_) deficiency in patients compared to controls and the UC-like colitis that develops in mice displaying biotin deficiency [11]. In addition, both biotin and niacin (i.e., vitamin B_3_) also possess specific anti-inflammatory properties targeted towards inflammatory bowel disease [12].

In Italy, there exists a readily available nutraceutical compound consisting of a combination of *H. erinaceus*, quercetin, berberine, niacin, and biotin (i.e., Enteroflegin^®^, Fenix Pharma SOC.COOP.P.A, Rome, Italy). This compound has garnered clinical and pre-clinical evidence supporting its efficacy in gut diseases. In detail, in human ex vivo tissue models of UC (as well as Crohn’s disease), it has been demonstrated that after two to three hours of exposure to such mucosal samples, the compound is capable of reducing the concentrations of cyclooxygenase-2 and tumour necrosis factor while simultaneously increasing the levels of the anti-inflammatory cytokine interleukin-10 [13]. From a clinical perspective, this compound has demonstrated promising efficacy in inducing clinical remission and reducing faecal calprotectin concentrations in patients with symptomatic uncomplicated diverticular disease [14]. This condition is, in fact, also characterised by an inflammatory component within the colonic environment [15].

However, despite the real-world use of this compound in Italy among patients with inflammatory bowel disease, standardised and robust data regarding effectiveness rates in patients with UC are lacking. Therefore, this retrospective multicentric study aimed to assess the real-life effectiveness of this compound as an add-on nutraceutical supplementation in patients with mild-to-moderate UC compared to standard conventional 5-ASA-alone treatment recommended by current clinical practice guidelines.

## 2. Materials and Methods

### 2.1. Study Design and Setting

This “HERICIUM-UC” study is a multicentre retrospective observational cohort two-arm study. It involved several Italian centres with good geographical representation (i.e., centres from the south, the centre, the islands, and the north of Italy), detailed and presented mainly in Appendix A, which routinely manage patients with UC. Data regarding variables and outcomes of interest were collected from the medical records of each centre and compiled into a shared online database. The data were collected in the first half of 2024 from all participating centres.

Each datum has been collected from records rigorously anonymised, omitting any information that could trace the variables back to the specific patient. Subsequently, the data have been processed in an entirely anonymous and strictly aggregated form. The study was presented and written following the checklist for retrospective cohort studies provided by the “Strengthening the Reporting of Observational Studies in Epidemiology” (STROBE) guidelines.

### 2.2. Inclusion/Exclusion Criteria and Study Groups

For patient inclusion in the analysis, several criteria were established: individuals with mild-to-moderate UC (as determined by a partial Mayo score, PMS [16] ranging from 2 to 7), aged between 18 and 70 years, undergoing conventional therapy solely with oral 5-ASA (i.e., mesalazine or sulfasalazine). Additionally, various exclusion criteria were delineated, encompassing the absence of probiotic/prebiotic/symbiotic/antibiotic supplementation/treatment for at least one month before the study’s baseline (neither during the study periods), pregnancy or lactation, a history of or active neoplasia, prior gastrointestinal surgery, current clinically significant infections (e.g., *Clostridioides difficile* or CMV), abnormal body mass index (namely patients with a BMI greater than 34 kg/m², classifiable as Class II obese, given the previously described risk of obese patients encountering more UC-related complications [17,18,19]), psychiatric disorders, and ongoing treatment with steroids, immunomodulators, biologics, or small molecules. Additionally, patients taking topical treatments for UC (e.g., 5-ASA or steroid enemas) were not considered for inclusion in the retrospective analysis. To this end, we selected patients who reported unwillingness to undergo topical therapy due to non-compliance.

The division into the two study groups, categorised as 5-ASA alone and the nutraceutical supplementation group, was based on an additional inclusion criterion: the additional consumption of Enteroflegin^®^ in addition to 5-ASA. This compound was required to adhere to a standard dosage regimen for all patients: two tablets per day of a single dosage (given that the commercial nutraceutical formulation was available in a single dosage form) for at least six months. For final inclusion in the study analysis, each of the two groups was required to have data available at baseline (T_0_), at three months after that (T_1_), and finally at six months later (T_2_). The absence of data at these time points excluded the patient from the study.

No exclusions were made for patients meeting the inclusion criteria from the available records (i.e., all consecutive patients were enrolled in the retrospective analysis within the study’s specified timeframe) at each centre to avoid excluding eligible patients, which could have increased potential risks of selection bias.

This nutraceutical product is, in accordance with current Italian regulations, duly registered and notified in the “Food Supplements Register” of the Italian Ministry of Health under code 126460. It is authorised for commercial sale throughout Italy without the need for a medical prescription and is freely purchasable by anyone. The composition of the compound (i.e., Enteroflegin^®^) is as follows: 525 mg of *H. erinaceus* powder (5% polysaccharides) and 225 mg of *H. erinaceus* as an extract (30% polysaccharides), 75 mg of quercetin titrated to 98%, 225 µg of biotin, 27 mg of niacin, and finally, 75 mg of *Berberis vulgaris* titrated to 97%. This combination, comprising solely *H. erinaceus*, berberine, and quercetin, is denoted as the “HBQ-Complex^®^”. Further technical specifications of the compound are detailed in Appendix B.

### 2.3. Study Variables

At baseline (T_0_), demographic and anthropometric variables of the patients were collected, including sex, age (in years), weight (in Kg), height (in m), body mass index (in Kg/m^2^), years since UC diagnosis, UC E-parameter according to Montreal classification (E1: proctitis, E2: left-colitis, E3: pancolitis) [20], alcohol consumption, and smoking status (active smoker or non-smoker). A patient was classified as an alcohol consumer if a self-reported weekly intake of no less than two alcoholic units was found.

At each time point (i.e., T_0_, T_1_, and T_2_), the faecal calprotectin level (in micrograms per gram) and the PMS were recorded from the medical records. At T_0_, the oral 5-ASA dose (in mg) was also recorded. The faecal calprotectin level had to be determined within seven days from the medical record date. According to the PMS, disease activity was classified as remission (PMS < 2), mild (2–4), moderate (5–7), or severe (>7) [16].

**Figure 1:**
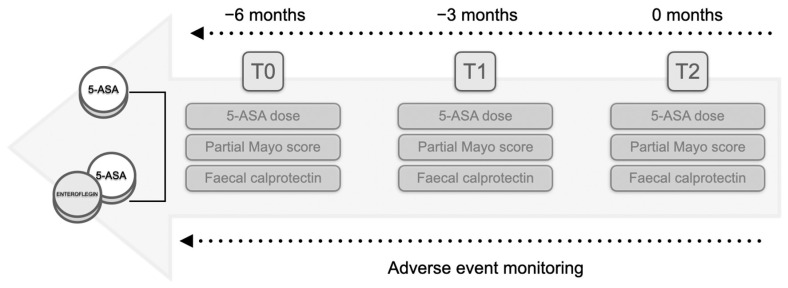
Flowchart of the study protocol. The study comprised two groups: patients undergoing therapy solely with derivatives of 5-aminosalicylic acid (5-ASA) and patients receiving add-on nutraceutical supplementation. Retrospective analysis involved the collection of the partial Mayo score alongside the faecal calprotectin levels and the 5-ASA dose at three months (T_1_) and six months (T_2_) from the initiation of treatment at T_0_ (a retrospective window of six months). Throughout the interval, any reported adverse events were sought in the records.

Lastly, according to the WHO classification, adverse events (AEs) reported in the records at all time points were also documented for safety assessment [21]. The study protocol is outlined in Figure 1.

### 2.4. Study Outcomes

The primary outcome of this study was clinical response at T_1_ and T_2_ (defined as a reduction in PMS compared to T_0_ of at least 2 points). The co-primary outcome was clinical remission at T_1_ and T_2_ (i.e., PMS < 2).

The secondary outcomes of the study included the real-world safety of the Enteroflegin^®^ compound (defined as the number of AEs recorded in the Enteroflegin^®^ group compared to the 5-ASA alone group) in our setting. Furthermore, through exploratory subgroup analysis, it was sought to ascertain whether the rates of clinical remission and response varied across the study periods according to the subgroups delineated by the study variables.

### 2.5. Statistical Analysis

Descriptive statistics were utilised to present the data. Continuous variables were displayed as the median, along with their respective interquartile range. The distribution of continuous variables was initially evaluated using a Kolmogorov–Smirnov test. A statistical significance threshold of *p* < 0.05 (strictly two-tailed) was adopted, thus establishing an alpha error of 0.05.

Various subgroup analyses were conducted to assess secondary outcomes, which were regarded as exploratory. Specifically, dichotomous independent variables (yes/no) for achieving clinical response/remission were examined. For this purpose, either a Mann–Whitney U-test for unpaired data or a Kruskal–Wallis test for dependent variable type (categorical with 2 or more degrees of freedom) were employed. For comparison between two categorical variables, an χ^2^ test (or Fisher’s exact test when applicable) was utilised.

Statistical analyses were conducted using IBM^®^ SPSS^®^ software (version 25, IBM Corp.©, Armonk, NY, USA), graphs were generated using GraphPad PRISM^®^ software (version 9.5.0, GraphPad Software LLC©, Boston, MA, USA), and sample size calculation was performed using G*Power software (version 3.1.9.6, Faul, Erdfelder, Lang, & Buchner, Dusseldorf, Germany).

## 3. Results

A total of 201 consecutive patients were finally included in the retrospective analysis. Out of them, 86 (42.7%) belonged to the 5-ASA-alone group and 115 (57.2%) to the group with nutraceutical supplementation. In the 5-ASA-alone group, there was a loss of 3 patients records at T_1_, while in the group with nutraceutical supplementation, there was a loss of 7 patients records at T_2_. Consequently, complete retrospective data were available from 83 to 108 patients per group, respectively, at all study times.

The clinical and demographic characteristics of the patients are summarised in Table 1. At baseline (T_0_), the population did not exhibit significant sample heterogeneity for the collected variables (*p* > 0.05, Table 1).

Across the entire sample, as shown in Table 1, 23 patients had recorded extra-intestinal manifestations associated with underlying UC in their medical records. In the 5-ASA-alone group, there was one case (1.16%) of erythema nodosum, one case (1.16%) of psoriasis, and nine cases (10.4%) of arthritis. In the nutraceutical supplementation group, there were two cases (1.73%) of psoriasis and ten cases (8.69%) of arthritis.

Regarding the primary outcome, the rates of clinical response in the 5-ASA alone group were 41% (34/83) and 60.2% (50/83) at T_1_ and T_2_, respectively. The rates of clinical remission, as co-primary outcome, instead were 16.9% (14/83) and 36.1% (30/83), respectively.

In the group with nutraceutical supplementation, the rates of clinical response were 49.6% (57/115) and 70.4% (76/108) at T_1_ and T_2_, respectively. Those of clinical remission were 30.4% (35/115) and 50.9% (55/108), respectively.

**Figure 2:**
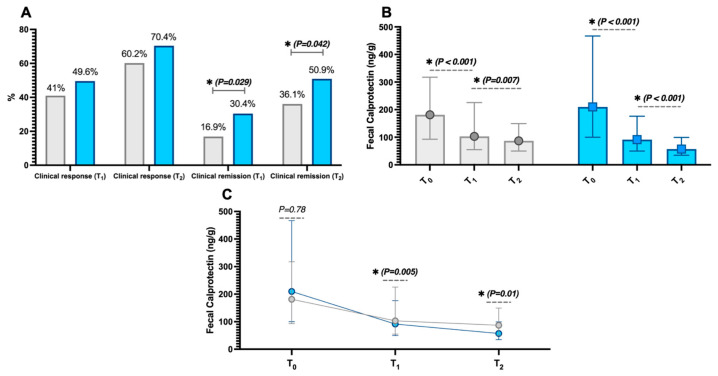
Clinical remission rates at three months (T_1_) and six months (T_2_) in the two study groups—those receiving 5-aminosalicylic acid (5-ASA) derivatives alone (grey) and those with nutraceutical supplementation (blue) from the baseline T_0_ (**A**). Additionally, the intragroup variations in faecal calprotectin (**B**) are shown, as well as the intergroup differences in calprotectin changes at different study time points (**C**). Significant *p*-values (i.e., <0.05) are highlighted in bold and marked with an asterisk (*). The data in Figures (**B**,**C**) are presented as median (interquartile range).

While the rates of clinical response, both at T_1_ (*p* = 0.231) and T_2_ (*p* = 0.143), did not show significant differences between the groups, conversely, those of clinical remission were different at both time points (*p* = 0.029 and *p* = 0.042, respectively, Figure 2A).

In support of these variations, faecal calprotectin levels significantly decreased during the follow-up period in both groups (*p* < 0.05, refer to Figure 2B). Additionally, observing the intergroup differences in faecal calprotectin variations, there were no differences between the two groups at baseline (*p* = 0.78). However, the decrease in calprotectin was more pronounced in patients with nutraceutical supplementation at both T_1_ (*p* = 0.005) and T_2_ (*p* = 0.01), as shown in Figure 2C. The dosages of 5-ASA were not different between the groups at both T_1_ and T_2_, indicating that there were no prevalent increases or decreases in dosages in either group (*p* > 0.05).

According to the records, no AEs emerged in either group.

## 4. Discussion

In this retrospective multicentre cohort observational study, we observed that adding a multi-compound nutraceutical *H. erinaceus*-based supplementation (i.e., Enteroflegin^®^) to standard oral 5-ASA may lead to better performance in achieving clinical remission at 3 (30.4% vs. 16.9%) and 6 (50.9% vs. 36.1%) months from the start of treatment compared to using 5-ASA alone in patients with mild-to-moderate UC according to PMS criteria [16]. As expected, given the nutraceutical nature of the supplementation add-on, we did not observe any alteration in the AEs profiles compared to the standard of care in the safety analyses. At the same time, as an objective control, this compound has been able to achieve a more pronounced reduction in faecal calprotectin levels over the retrospective observation period compared to the standard of care.

To the best of our knowledge, no other human studies have evaluated the therapeutic potential of this compound as an add-on or as a single agent in the context of UC. The only available evidence pertains to symptomatic uncomplicated diverticular disease, where it has already demonstrated a good clinical remission rate (approximately 20% at six months in a large sample of over three hundred patients) and a reduction in faecal calprotectin levels [14].

It is not uncommon for the incorporation of a probiotic nutraceutical supplement alongside 5-ASA to potentially yield a reduction in a biochemical indicator of UC disease activity, such as faecal calprotectin, in studies akin to ours. An instance is the trial conducted by Jiang et al. [22], which encompassed a smaller cohort (i.e., 180 UC patients) divided into two groups (one receiving only 5-ASA and the other receiving an additional *Bifidobacterium*-based probiotic supplement). In this trial, the inclusion of the nutraceutical supplement led to a more notable decrease (respect to 5-ASA alone group) in calprotectin levels after two months of treatment. However, other probiotics (e.g., *Escherichia coli* Nissle 1917) have even demonstrated equivalence to 5-ASA (i.e., mesalazine) in clinical trial settings [23].

We regard the identification and investigation of nutraceutical supplementation, alongside the recognition of functional foods, as a priority for the management of inflammatory bowel diseases. These conditions are chronic and lifelong, and as of today, we lack a definitive therapy that can ensure sustained disease clearance over time. Even surgical intervention for UC, when indicated with its curative intent, can be complicated by new chronic complications (such as inflammatory disorders of the pouch) [24].

For mild-to-moderate UC, 5-ASA therapy remains the gold standard treatment [2,3], both orally and topically. However, a certain proportion of patients are intolerant or non-compliant to the use of topical therapy and prefer exclusive oral therapy [25]. Nevertheless, it is known that combination therapy (oral and topical 5-ASA) is more effective than monotherapy in inducing UC clinical remission [26]. However, despite this, it should be emphasised that even though the response to 5-ASA exhibits a clear dose-dependency and presents an optimal safety profile compared to placebo, from the most robust meta-analyses available, a certain proportion of patients, even exceeding 50%, fail to achieve clinical remission with the use of 5-ASA [27]. For this reason, over time, efforts have been made to identify add-on therapies to the standard one that could enhance the efficacy of 5-ASA to avoid switching to advanced therapies in the UC setting. However, to date, there is low-certainty evidence regarding the role of probiotics in inducing clinical remission in active UC compared to placebo, either as a standalone treatment or in combination with 5-ASA [5]. Initial data suggest that the combination of prebiotics and probiotics (i.e., symbiotics) may have a certain role as a complementary therapy in UC, within the limits, once again, of the need for further robust studies [28].

The complete mechanism by which this combination compound provides the observed benefits, both in ex vivo contexts [13] and in the present clinical study, is not yet fully understood. Several pieces of evidence are available in preclinical models for the individual components of the compound. For instance, *H. erinaceus* has shown, primarily through its polysaccharide components, to improve experimental colitis through two main mechanisms: regulation of oxidative stress and down-regulation of nuclear factor kappa B (NF-κB) [8]. NF-κB is a well-known modulator of tumour necrosis factor, which *H. erinaceus* has demonstrated to down-regulate in combination with the other components of Enteroflegin^®^ in human inflammatory bowel disease ex vivo models [13]. In addition, *H. erinaceus* can influence the balance of the gut microbiota in humans by selecting for short-chain fatty-acids-producing bacteria [29]. It is well known that these acids, which also have immunomodulatory functions with anti-inflammatory effects, primarily including acetate, propionate, and butyrate, are significantly reduced in the intestinal microenvironment of inflammatory bowel diseases, leading to a reduction in intestinal epithelial barrier integrity [30,31,32]. Certainly, this speculation would need validation through specific observations in dedicated trial studies conducted with gut microbiota analysis. Our study, being observational and real-life, strictly adheres to the European guidelines for the management of mild-to-moderate UC [2]. Currently, these guidelines exclude the recommendation to analyse both these faecal mediators (i.e., short-chain fatty acids) and the gut microbiota from the diagnostic–therapeutic process. However, future studies may examine the impact of the compound under study on these parameters, particularly considering the well-established probiotic properties of *H. erinaceus* [8,33,34], quercetin [35], and berberine [36,37].

An additional interesting finding (considering the significant risk of colorectal cancer in UC patients [38]) is that numerous studies on cellular models of colonic cancer (primarily CT-26, HT-29, HCT-116 cells) have outlined the potential of this fungus to positively intervene in the pathogenesis of colorectal cancer through various mechanisms [8]. These include, for example, increasing the activity of natural killer cells and macrophages, reducing angiogenesis, and intervening in critical pathways such as the AKT/mTOR pathway [8].

Regarding the other components of the combination compound, quercetin has similarly been demonstrated to improve microbiota diversity and disease severity affecting the colon in mouse models of experimental colitis [39]. Furthermore, for berberine, a small pilot phase I trial is available, providing evidence of the benefit of improving tissue inflammation grade (evaluated using the Geboes score) in patients with remission of UC in maintenance with 5-ASA [40].

From the results of this study, there also emerges a greater capacity of this compound to achieve clinical remission compared to clinical response both at T_1_ and T_2_. In fact, at both time points, the rates of clinical response did not differ from those of 5-ASA alone. In our view, these data should be interpreted purely by mathematical considerations. Starting from a large portion of the sample (i.e., 120, 59.7%) that had mild disease at baseline (i.e., with a PMS of 2–4), and considering that the standardised [41] requirement for achieving remission was a PMS < 2, it is easier in this setting, compared to moderate disease (i.e., PMS 5–7), to achieve the clinical outcome of remission rather than response (which would require a reduction of at least two points from baseline). Nonetheless, the most clinically relevant outcome remains remission, as it pertains to clear UC disease activity control and is associated with better long-term outcomes [6].

This study has several strengths: its multicentre nature, the large sample size (provided with statistical power sufficient to at least detect medium-to-large effect sizes), the real-world nature allowing the assessment of this compound’s performance in actual clinical practice, the inclusion of an objective biomarker such as faecal calprotectin, the use of a validated system to assess clinical response and remission (i.e., PMS), and finally, the two-arm comparison with the standard of care (i.e., 5-ASA). 

However, this study also has several limitations, including its retrospective and uncontrolled nature, the lack of serological and endoscopic/histological evaluations of disease activity, and the exclusive focus on induction of remission rather than maintenance thereof. In addition, within the constraints of the absence of endoscopic evaluations, which are not feasible in our retrospective and observational setting as they are not routinely indicated for all patients, especially those with non-severe activity, faecal calprotectin presents several vicarious opportunities. Indeed, multiple studies have outlined how faecal calprotectin can serve as a non-invasive surrogate marker of endoscopic and histological disease activity [42,43,44]. Despite this, the more pronounced reduction observed in the group with nutraceutical supplementation offers another consideration. It is also known that faecal calprotectin, in settings of good disease control, has predictive power for subsequent disease flare-ups, and that lower calprotectin levels correspond to a concurrent reduction in this risk [45].

Therefore, the reduction in faecal calprotectin is an extremely relevant target, with the advantage of being non-invasive, easily repeatable, and it is not coincidental that it is highlighted by international consensus as a goal in the treat-to-target strategy for UC [6]. In other words, in a real-world and observational setting like ours, a strict and frequent endoscopic follow-up during the study period and the availability of baseline endoscopies for the entire sample within comparable timeframes was impractical, as clinical practice did not indicate it [46]. However, this aspect should be addressed in future longitudinal studies by integrating endoscopic outcomes to evaluate the endoscopic effectiveness of nutraceutical supplementation as an add-on to 5-ASA, precisely to determine if it makes a difference in achieving mucosal healing.

Additionally, the absence of AEs and therefore the normality of haemato-chemical tests for renal and hepatic function during follow-up provide reassurance regarding potential hepatotoxic or nephrotoxic AEs associated with this add-on supplementation.

Additionally, the sample sizes of the two study groups are not entirely equivalent. This discrepancy is partly explained by the difficulty of identifying a larger sample of control patients on exclusive oral 5-ASA treatment with confirmed non-compliance to topical treatment due to the stringent inclusion criteria set for this study. Fortunately, such patients do not represent most of those with mild-to-moderate UC [47,48]. Nevertheless, the two groups had no significant sample heterogeneity, as demonstrated in Table 1.

Lastly, another limitation is the inability to conduct robust subgroup analyses, albeit exploratory, on potential differences in the outcomes of interest regarding the presence or absence of extra-intestinal manifestations, due to the insufficient sample size of such subgroups in our study.

## 5. Conclusions

In conclusion, these data suggest a real-world therapeutic potential of Enteroflegin^®^ when added to 5-ASA in inducing remission in mild-to-moderate UC. Additionally, from the data in our setting, it appears that the reduction in faecal calprotectin is more pronounced when this nutraceutical is added to the standard of care.

This underscores the need for a well-powered randomised controlled trial to assess the efficacy and safety rates of this compound comprehensively.

## Figures and Tables

**Table 1:** Clinical–demographic characteristics of the sample at the study baseline divided into the groups included in the retrospective analysis.

Variable	5-ASA Alone Group (*N* = 86)	5-ASA plus Nutraceutical Supplementation (*N* = 115)	*p*-Value ^1^
**Age** (years)	40 (39–46)	44 (42–47)	0.642
**Gender** Male Female	41 (47.7%) 45 (52.3%)	66 (57.4%) 49 (42.6%)	0.172 ^3^
**UC diagnosis** (years)	7 (2–17)	7 (2–17)	0.223
**BMI** (Kg/m^2^)	23 (22.5–24.1)	23.9 (23.5–24.6)	0.404
**Alcohol status** ^2^ (yes)	10 (11.62%)	18 (16.65%)	0.472 ^3^
**Smoking status** (yes)	22 (25.58%)	27 (23.47%)	0.712 ^3^
**Montreal classification** E1 (ulcerative proctitis) E2 (left-sided UC) E3 (ulcerative pancolitis)	15 (17.4%) 51 (59.3%) 20 (23.3%)	18 (15.7%) 71 (61.7%) 26 (22.6%)	0.926
**5-ASA status** Mesalazine Sulfasalazine	84 (97.7%) 2 (2.3%)	110 (95.7%) 5 (4.3%)	0.439
**5-ASA dose** (mg)	3200 (2400–3600)	3600 (2400–3600)	0.364
**5-ASA dose class** Standard (<3.6 g) High (≥3.6 g)	46 (53.5%) 40 (46.5%)	50 (43.5%) 65 (56.5%)	0.16 ^3^
**EIM** (yes)	11 (12.79%)	12 (10.43%)	0.863 ^3^
**PMS**	4 (3–6)	4 (3–6)	0.986
**PMS class** Mild disease Moderate disease	51 (59.3%) 35 (40.7%)	69 (60%) 46 (40%)	0.921 ^3^
**Faecal calprotectin** (µg/g) ^4^	181.5 (92.7–317.5)	210 (100–467)	0.78

Continuous variables are expressed as median (interquartile range), while categorical and ordinal variables are presented as frequencies. ^1^ The *p*-value is calculated to assess potential heterogeneity among the variables considered within the two study groups. ^2^ An individual was classified as an alcohol consumer if a self-reported weekly intake of no less than two alcoholic units was identified. ^3^ This analysis was conducted using the χ^2^ test (or Fisher’s exact test when applicable). ^4^ Normal value < 50 µg/g. Acronyms: BMI: body mass index; UC: ulcerative colitis; 5-ASA: 5-aminosalicylic acid; EIM: extra-intestinal manifestations; PMS: partial Mayo score.

## Data Availability

The original contributions presented in the study are included in the article, further inquiries can be directed to the corresponding author.

## Appendix A

The **HERICIUM-UC study group**: **Monica Carta** (Gastroenterology Division, SS Annunziata Hospital, Sassari, Italy); **Marco Astegiano** (Gastroenterology Division, Presidio San Giovanni Antica Sede, Torino, Italy); **Paola Cognein** (Gastroenterology Division, Villa Scassi, Genova, Italy); **Berardino D’Ascoli** (Gastroenterology Division, Madonna delle Grazie Hospital, Matera, Italy); **Donato Iannuzziello** (Gastroenterology Division, Mater Dei Hospital, Bari, Italy); **Antongiulio Bucci** (Gastroenterology and Digestive Endoscopy Division, San Paolo Hospital, Bari, Italy); **Tammaro Maisto** (Gastroenterology Division, S. Maria della Pietà Hospital, Casoria, Italy); **Domenico Napoletano** (Digestive Endoscopy Division, Pineta Grande Hospital, Castel Volturno, Italy); **Giovanni Lombardi**, **Marta Patturelli** (Gastroenterology and Digestive Endoscopy Division, Cardarelli Hospital, Naples, Italy); **Giuliana Vespere**, **Silvia Sedda** (Gastroenterology and Digestive Endoscopy Division, Ospedale del Mare, Naples, Italy); **Roberto Lamanda**, **Nicola Imperatore** (Gastroenterology and Digestive Endoscopy Division, Santa Maria delle Grazie Hospital, Pozzuoli, Italy); **Rodolfo Sacco** (Gastroenterology and Digestive Endoscopy Division, University Hospital of Foggia, Foggia, Italy); **Antonella Scarcelli** (Gastroenterology and Digestive Endoscopy Division, AST Pesaro, Urbino, Italy); **Sergio Cavenati** (Territorial Gastroenterology, Treviglio, Italy); **Antonio Loprete Morabito** (Digestive Endoscopy Division, Tropea ASP Hospital, Vibo Valentia, Italy); **Ileana Luppino** (Gastroenterology and Digestive Endoscopy Division, Cosenza Hospital, Cosenza, Italy); **Chiara Sterpi** (Digestive Endoscopy Division, Ospedali Riuniti, Livorno, Italy); **Luca Sediari** (Gastroenterology Division, Santa Maria della Misericordia Hospital, Perugia, Italy); **Marco Bassi** (Gastroenterology Division, AUSL, Bologna, Italy); **Rita Ortenzi, Lorenza Tifi** (Gastroenterology Division, USL Umbria 1, Italy); **Antonio Romano** (Territorial Gastroenterology, Pisa, Italy); **Rocco Ranaldo** (Internal Medicine Division, Mazzolani Vandini Argenta Hospital, AUSL Ferrara, Ferrara, Italy); **Antonio Bordonaro** (Gastroenterology and Digestive Endoscopy Division, Umberto I Hospital, Enna, Italy); **Corrado Selvaggio** (Gastroenterology Division, Ospedale Maggiore di Modica, Modica, Italy); **Andrea Cocco** (Gastroenterology and Digestive Endoscopy Division, Sandro Pertini Hospital, Rome, Italy); **Giuseppe Pianese** (University Gastroenterology and Digestive Endoscopy Division, Santa Maria Goretti Hospital, Latina, Italy); **Enrica Evangelista** (Gastroenterology Outpatient Division, Frosinone, Italy); **Maurizio Giovannone**, **Giuseppina Vincoli** (Gastroenterology Division, San Camillo De Lellis Hospital, Rieti, Italy); **Roberto Faggiani**, **Rita Monterubbianesi** (Gastroenterology and Digestive Endoscopy Division, San Camillo Forlanini Hospital, Rome, Italy).

## Appendix B

The manufacturing specifications of the multi-compound nutraceutical are as follows: The 5% polysaccharide *H. erinaceus* component powder, directly derived from the fungal sporophorum, exhibits macroscopic characteristics of a fine brown powder. The polysaccharide content of the powder is 5%, as determined by ultraviolet–visible spectroscopy. The *H. erinaceus* 30% polysaccharide extract is derived from the fungal sporophorum and contains 10% maltodextrin as an excipient. The solvent used for extraction is 100% water, with an extraction ratio of 10:1. The 30% polysaccharide content is evaluated using ultraviolet-visible spectroscopy. The macroscopic appearance is a fine yellow-brown powder almost entirely soluble in water. Both extracts contain polycyclic aromatic hydrocarbons at levels ≤50 parts per billion and benzo(a)pyrene at ≤10 parts per billion, as measured by gas chromatography-mass spectrometry. The apparent density of both compounds ranges from 0.4 to 0.7 g/mL. Sieve analysis shows 100% passing through an 80 mesh. The loss on drying and total ash content is ≤10%. The total plate count is ≤1000 colony-forming units per gram. Heavy metals are present at ≤10 mg/Kg in both compounds. The 5% powder and the 30% polysaccharide extract are free from ethylene oxide and irradiation and contain less than 20 parts per million of gluten. According to the datasheet, the shelf life is 24 months if stored in a tightly closed container, protected from moisture, light, and oxygen.
